# Healthcare costs and effects of post-COVID-19 condition in Canada

**DOI:** 10.14745/ccdr.v49i10a03

**Published:** 2023-10-01

**Authors:** Ellen Rafferty, Ali Unsal, Erin Kirwin, Institute of Health Economics, Edmonton, Department of Medicine, Faculty of Medicine and Dentistry, University of Alberta, Edmonton, Health Organisation, Policy, Economics, School of Health Sciences, University of Manchester, Manchester, United Kingdom

**Keywords:** post-COVID condition, economic burden, healthcare costs, costing analysis, quality-adjusted life years, COVID-19

## Abstract

**Background:**

As evidence of the long-term health impacts of coronavirus disease 2019 (COVID-19) continues to grow across Canada, a key concern is the costs and health impacts of post-COVID-19 condition (PCC), especially while the healthcare system remains under substantial strain. The objective of this study is to estimate healthcare costs and quality-adjusted life year (QALY) decrements per PCC case and per acute COVID-19 case by vaccination status.

**Methods:**

First, we conducted a rapid review of the literature to estimate 1) the probability of developing PCC following COVID-19 infection by vaccination status, 2) the probability of each condition commonly associated with PCC, 3) healthcare costs and QALY decrements associated with each condition and 4) the number of PCC cases currently in Canada. Second, using the data gathered from the literature, we built a tool to estimate the cost and QALY decrements per PCC and COVID-19 case.

**Results:**

Post-COVID-19 condition costs per COVID-19 case ranged from CAD 1,675 to CAD 7,340, and QALY decrements ranged between 0.047 to 0.206, in the first year following COVID-19 infection. Overall, individuals who were unvaccinated when they were infected had higher costs and QALY decrements. We estimated the total burden of PCC to the Canadian healthcare system based on PCC estimates up until spring 2023 would be between CAD 7.8 and CAD 50.6 billion.

**Conclusion:**

This article demonstrates the large potential health and economic burden of PCC for Canadians, and the importance of vaccination and other infection control strategies in reducing the longer-term costs and effects.

## Introduction

Concerns continue to grow about the long-term impacts of the coronavirus disease 2019 (COVID-19) pandemic. Post-COVID-19 condition (PCC) is often characterized by ongoing symptoms (12 weeks or longer) after the acute phase of COVID-19 infection ((1)). Symptoms commonly associated with PCC include fatigue, respiratory symptoms, adverse cardiovascular events, psychiatric and cognitive issues, along with other symptoms that impact everyday functioning. Post-COVID-19 condition symptoms can fluctuate over time and include those that persist over many months following acute COVID-19 or brand new symptoms that occur following initial recovery ((2)). A large retrospective matched cohort study examined risk factors for PCC in adults with confirmed COVID-19 who were propensity score matched to controls without COVID-19 ((3)). Among the cohort with confirmed COVID-19 infection, risk factors significantly associated with PCC included female sex, belonging to an ethnic minority, socioeconomic deprivation, smoking, obesity and a wide range of comorbidities; raising concerns about equity, as impacts of PCC differ among groups of the population.

Many studies have demonstrated a high prevalence of PCC, although the risk of PCC following COVID-19 diagnosis varies widely across studies (5%–80%) ((4)). Two systematic reviews ((5,6)) estimated that 63% to 84% of people with confirmed COVID-19 had symptoms four weeks after either diagnosis or hospitalization and 46% to 56% experienced symptoms after 12 weeks. However, it appears that prevalence decreases with time (i.e. fewer people report symptoms at six to nine months compared to at three months), which may indicate that some people with PCC could recover over time. Studies also suggest those with a higher severity of acute infection (e.g. those hospitalized) may be at a higher risk of PCC compared to people who had milder acute illness ((7,8)). In 2021, in Canada, there were estimated to be 150,000 individuals with PCC, based on a rapid systematic review (9). A Canadian survey of individuals with confirmed or suspected PCC found close to 50% of respondents had symptoms following acute COVID-19 infection for longer than 11 months ((10)).

Post-COVID-19 condition is associated with increased healthcare utilization, but there is little evidence estimating health system cost and quality of life impacts. A community-based matched cohort study of Ontario adults, both with and without prior polymerase chain reaction-confirmed COVID-19, estimated healthcare utilization 56 days after initial infection ((11)). Using a composite measurement, which included home care, long-term care, hospitalization, outpatient, and emergency department visits, they found healthcare utilization was 11% higher in individuals that tested positive for COVID-19 compared to those that did not, leading to an additional 1.4 healthcare encounters per person year ((11)). However, to date, how these additional healthcare encounters may translate into increased healthcare system costs in Canada, in the short and medium term has not been explored. Moreover, with the variety of symptoms associated with PCC, very little is known about how PCC impacts quality of life in Canada. These estimates are important to help evaluate the economic benefits associated with preventing COVID-19 and PCC, as well as treatments for PCC.

The overall objective of this study is to provide cost and health-related quality of life estimates on PCC and specifically to estimate 1) healthcare costs associated with a PCC case and the cost of PCC per acute COVID-19 case as well as total healthcare cost burden of PCC and 2) quality-adjusted life year (QALY) decrement per PCC case and QALY decrement due to PCC per acute COVID-19 case.

## Methods

This analysis built on work by Mulberry *et al.* ((12)) that estimated the healthcare cost associated with PCC as part of a larger economic evaluation of vaccine roll-out strategies. We updated this analysis to produce estimates of the healthcare cost and QALY decrements associated with PCC per COVID-19 case disaggregated by vaccination status.

In the first step, we conducted a rapid review to 1) estimate the probability of developing PCC following COVID-19 infection and by vaccination status, 2) estimate the number of PCC cases currently in Canada, 3) identify the symptom classes and conditions most commonly associated with PCC, and the probability they will develop, and 4) determine the healthcare costs and QALYs associated with each of the PCC symptom classes.

We conducted the rapid literature review using the PubMed/MEDLINE and Google Scholar databases. Search terms across the four topics included COVID-19, PCC, probability, incidence, symptoms, cost, QALY and Canada, and derivations of these keywords. For a full list of the keywords see the **Appendix A**, **Table A1**. Our search did not include non-English-language articles.

This review did not include a formal quality appraisal, as the studies we needed to conduct the analysis were very diverse in methods. However, when selecting articles for inclusion in the review we prioritized the PCC symptom class, QALY, and costing studies to be included in the analysis based on four factors: 1) Canadian specific data; 2) sample size; 3) the consistency of the reported condition with reported PCC symptoms, and 4) how recently the data were collected. Based on these prioritization criteria, we used the results from the Canadian COVID-19 Antibody and Health Survey to identify symptom class, as it was a large ongoing survey of the Canadian population and provided information across symptoms for PCC. Moreover, since the first three topic areas were focused on COVID-19, we included only papers published after December 2019 in this part of the review.

In the second step, using the data gathered from the literature, we conducted a costing analysis of the cost per PCC case and PCC-associated cost per COVID-19 case. Input parameters derived from the literature included 1) the total number of patients who experienced at least one PCC symptom in Canada by spring 2023, 2) the likelihood of becoming a PCC case stratified by vaccination status, as well as 3) the probabilities, costs and QALY decrements for each of the most common PCC symptom classes.

We reviewed several symptom classes associated with PCC, including, chronic fatigue, cognitive conditions (e.g. brain fog), diabetes, psychiatric conditions (e.g. depression/anxiety), chronic liver disease, chronic kidney disease, adverse cardiovascular events and respiratory disease. We selected the four most common symptom classes (chronic fatigue, cognitive conditions, psychiatric conditions and respiratory disease) for inclusion in the analysis. Costs were captured in 2022 Canadian dollars (CAD) and effects were measured in QALY decrements. For a full list of input parameters see **Table 1**.

**Table 1:** Input parameters

Variable	Estimate		Source, year publication (reference)
Symptom classes	Probabilities (%)		
	Non-overlapping	Overlapping	
Chronic fatigue	42.99	72.1	Health Infobase, 2022 ((13))
Psychiatric conditions	14.43	24.2	
Cognitive conditions	19.62	32.9	
Respiratory disease	22.96	38.5	
**Likelihood of becoming a PCC case (by vaccination status)**			
Unvaccinated	41.8	N/A	Azzolini *et al.*, 2022 ((14))
1^st^ dose	30.0		
2^nd^ dose	17.4		
3^rd^ dose	16.0		
**Health related quality of life decrements**			
Chronic fatigue	0.36		Versteegh *et al.*, 2016 ((15))
Psychiatric conditions	0.21		Steensma *et al.*, 2016 ((16))
Cognitive conditions	0.12		Song *et al.,* 2022 ((17))
Respiratory disease	0.37		Van Wilder *et al.,* 2019 ((18))
**Costs (Canadian dollars)**			
Chronic fatigue	12,753		Jason *et al.,* 2008 ((19))
Psychiatric conditions	4,123		Chiu *et al.,* 2017 ((20))
Cognitive conditions	9,939		Zhu *et al.,* 2013 ((21))
Respiratory disease	10,641		Bonafede *et al.,* 2011 ((22))
**Other variables**			
Number of patients with at least one PCC symptom	Low: 0.74 million Medium: 2.02 million High: 2.88 million		Health Infobase, 2023 ((23)) Health Infobase, 2023 ((24)) Statistics Canada, 2023 ((25))

Abbreviations: N/A, not applicable; PCC, post-COVID-19 condition (COVID-19, coronavirus disease 2019)

We estimated costs and QALY decrements of PCC under two scenarios; 1) non-overlapping symptomology; and 2) overlapping symptomology. In the non-overlapping symptomology scenario, we estimated the healthcare costs and QALY decrements assuming PCC symptoms were mutually exclusive. Therefore, in this calculation, someone diagnosed with PCC would have costs and QALY decrements associated with only one symptom class of PCC (see Equation 1). To calculate the probability of an individual with PCC having a specific symptom class, we took the probability of developing each symptom class from the literature and weighted them to sum to one.

In comparison, in the overlapping symptomology scenario individuals infected with COVID-19 had a certain risk of developing each of the PCC symptom classes, and therefore, could have the costs and QALY decrements associated with more than one symptom class (see Equation 2). In this case, the overall probability of PCC was the raw sum of the probability of having each symptom class PCC diagnosis as derived from the literature. We made this assumption because there is no information available in the literature on joint probability by symptom class, or how this impacts healthcare costs and QALY decrements. For example, this assumes that an individual with PCC that causes chronic fatigue and cognitive conditions will have expected healthcare costs and QALY decrements equal to the sum of the costs and QALY decrements of these two conditions individually multiplied by the probability of each condition. All analyses were conducted using a one-year time horizon; however, the tool allows for longer-term analysis of the costs and effects of PCC. For analyses past one year, we apply an annual discount rate of 1.5% to both costs and QALY decrements, following Canadian guidance ((26)). The full tool is available to view and download.

**Equation 1:** Cost and quality-adjusted life year decrements per post-COVID-19 condition case and post-COVID-19 condition-associated cost and quality-adjusted life year decrements per acute COVID-19 case in the non-overlapping scenario.

*Cost per PCC case* = ∑*_s_* (*p_n_s_* × *c_s_*)

*Cost of PCC per acute COVID-19 case by vaccination status* = *p_pcc_v_* x ∑*_s_* (*p_n_s_* × *c_s_*)

*QALY loss per PCC case* = ∑*_s_* (*p_n_s_* × *u_s_*)

*QALY loss of PCC per acute COVID-19 case by vaccination status* = *p_pcc_v_* x ∑*_s_* (*p_n_s_* × *u_s_*)

**Equation 2:** Cost and quality-adjusted life year decrements per post-COVID-19 condition case and post-COVID-19 condition-associated cost and quality-adjusted life year decrements per acute COVID-19 case in the overlapping scenario.

*Cost per PCC case* = ∑*_s_* (*p_o_s_* × *c_s_*)

*Cost of PCC per acute COVID-19 case by vaccination status* = *p_pcc_v_* x ∑*_s_* (*p_o_s_* × *c_s_*)

*QALY loss per PCC case* = ∑*_s_* (*p_o_s_* × *u_s_*)

*QALY loss of PCC per acute COVID-19 case by vaccination status* = *p_pcc_v_* x ∑*_s_* (*p_o_s_* × *u_s_*)

Where:

*p_n_s_* stands for the probabilities of symptom classes under the non-overlapping scenario (i.e. weighted probabilities sum to one)

*p_o_s_* stands for probabilities of symptom classes under the overlapping scenario (i.e. raw sum of probabilities)

*c_s_* stands for costs associated with symptom classes

*u_s_* stands for utility decrements associated with symptom classes

*p_pcc_v_* stands for probability of becoming a PCC case following acute COVID-19 by vaccination status

*s* stands for array of all symptoms classes, s = 1….S

As we described in Equation 1, we first calculated the cost and QALY decrement per PCC case and then we applied the probability of having PCC by vaccination status to estimate the PCC-associated cost and QALY decrement per acute COVID-19 case under the non-overlapping scenario. For Equation 2, since we assumed PCC symptoms may overlap, we estimated the cost and QALY loss from each PCC case would equal the sum of all symptom probabilities multiplied by the costs and QALYs of those symptoms. We then apply the probability of having PCC by vaccination status, to estimate the PCC-associated cost and QALYS decrement per acute COVID-19 case.

Finally, to estimate total costs associated with PCC in Canada we multiplied the costs per PCC case with the estimated number of PCC cases that have occurred in Canada as of spring 2023. Due to high variability in the literature, we used a range of values for the number of PCC cases. The low estimate was calculated by multiplying confirmed cases of PCC as reported by the Government of Canada as of August 1, 2023 ((23)), in combination with the lower bound of the confidence interval for the percent of COVID-19 cases that result in PCC ((24)). The middle value was calculated using 2023 Canadian population estimates ((25)), in combination with the percent of people reporting testing positive for COVID-19 on rapid antigen or polymerase chain reaction test and the percent of adults reporting PCC following infection ((24)). Finally, the high value was calculated using the 2023 Canadian population estimates ((25)), as well as the percent of people either reporting testing positive or having suspected infection, and the high bound of the confidence interval of the percent of cases reporting long-term symptoms ((24)).

## Findings

The results of the non-overlapping symptomology scenario indicate that the costs and QALY decrements per PCC case are CAD 10,471 and 0.29 QALYs within a year, respectively. While the overlapping symptomology scenario indicates higher costs and utility decrements per year associated with PCC, of CAD 17,559 and 0.49, respectively. Based on the estimate on a range of scenarios of the number of Canadians with PCC from CAD 0.74 million to 2.88 million in spring 2023, the total burden to the Canadian healthcare system for one year range between CAD 7.8 and CAD 30.2 billion (middle value: CAD 21.2 billion) in the non-overlapping scenario. Yearly costs of PCC in the overlapping scenario were even higher, ranging from CAD 13.0 to CAD 50.6 billion (middle value: CAD 35.5 billion).

**Figure 1:**
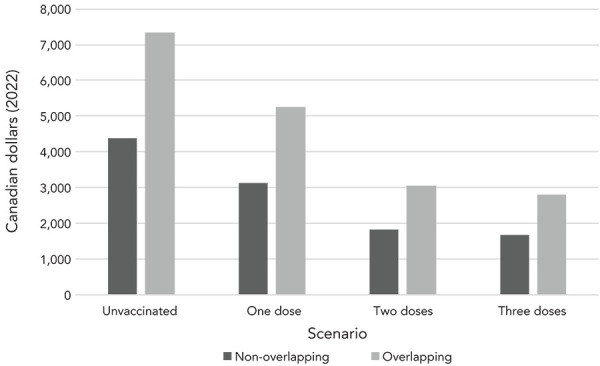
Annual post-COVID-19 condition cost (in 2022 Canadian dollars) per COVID-19 case by symptomology scenario and vaccination status Abbreviation: COVID-19, coronavirus disease 2019

**Figure 2:**
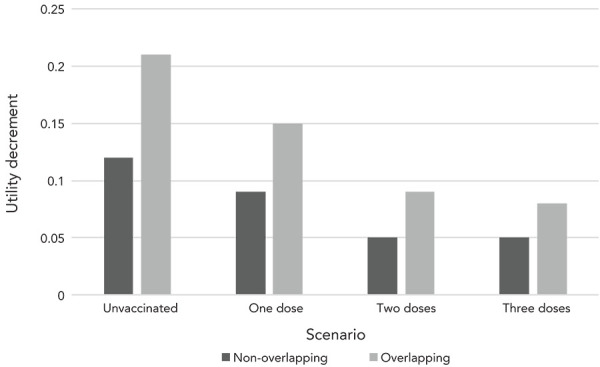
Annual post-COVID-19 condition quality-adjusted life years decrement per COVID-19 case by symptomology scenario and vaccination status Abbreviation: COVID-19, coronavirus disease 2019

Vaccination had a substantial impact on PCC-associated costs and QALY decrements per acute COVID-19 case calculated under the non-overlapping scenario, presented in **Figure 1** and **Figure 2**. Post-COVID-19 condition cost and QALY decrements per COVID-19 case were 1.4 times, 2.4 times and 2.6 times greater for those who were unvaccinated compared to those vaccinated with one, two and three doses, respectively. **Appendix B** (Scenario analysis findings) provides detailed numeric results for both the overlapping and non-overlapping scenarios.

Based on the assumption of overlapping symptoms, PCC-associated costs and QALY decrements were at least one and a half times any of the estimates from the non-overlapping scenario. Considering that the majority of the Canadian population has two doses of vaccination (with an expected PCC-associated cost per COVID-19 case of CAD 1,822 in the non-overlapping scenario and CAD 3,055 in the overlapping scenario), we can infer that PCC costs and QALY decrements per acute COVID-19 case in the overlapping scenario are 1.7 times the non-overlapping scenario.

## Discussion

We examined the cost and QALY impact of PCC in Canada under two scenarios, both of which emphasize the importance of vaccination. Having at least two doses of COVID-19 vaccine was associated with a large decrease in PCC costs following an acute COVID-19 infection. In comparison, while booster doses still reduced PCC costs and improved QALYs, the marginal benefit was lower. However, as COVID-19 vaccine immunity wanes over time the benefits of booster doses may increase, and it is therefore important to continuously update estimates on the impact of vaccination on PCC.

Both scenarios demonstrate that PCC-associated costs and QALY decrements can be tremendous, ranging from CAD 1,675 to CAD 7,340 per acute COVID-19 case per year. Using current estimates of PCC in Canada, we assessed the healthcare costs associated with these conditions between CAD 7.8 and CAD 50.6 billion per year. Without more information on if and how PCC patients are seeking healthcare, along with PCC severity estimates and recovery times, it is hard to know if the estimates presented here are high or conservative. Therefore, these estimates should be adjusted as more information becomes available in the literature. Alternatively, if individuals with PCC do not seek care for these conditions, or have trouble receiving care, this could manifest in future costs to the healthcare system as conditions worsen. In this analysis we did not capture productivity losses due to PCC, such as absenteeism, presenteeism, or early retirement. Previous research from the United States demonstrates that the productivity losses from PCC may result in economic losses in that country ranging from 101 to USD 403 billion ((27)).

## Limitations

This analysis has several limitations. First, there is a large degree of uncertainty in our results, and these estimates should be updated as more details emerge about the probability of developing PCC, as well as how individuals with PCC seek care and the healthcare costs and QALY decrements associated with the symptoms of PCC. In particular, the nature of the available data meant we needed to make assumptions about the relationship between PCC symptom probabilities, and the costs and outcomes associated with those symptoms, as observed in the differences in the overlapping and non-overlapping estimates. Second, while we searched the literature for the most updated costs and disutility values for the relevant symptom classes, some of the values have not been updated recently, and therefore may not represent current costs and outcomes. However, this tool is easily updated as newer costing and disutility values become available, and there is more information on the risk of PCC and number of Canadians impacted. Third, the literature on development of, and recovery from, PCC is constantly shifting as more data on this population become available. For example, the evidence on recovery from PCC is still developing. This is why we chose to focus on yearly cost estimates, rather than predicting further into the future. Prediction information should be incorporated into this tool as it becomes available to provide accurate information for decision-making. Finally, we provide only a mean-based estimate under two scenarios with ranges around the number of PCC cases in Canada, and future work could take on a more Bayesian approach to the uncertainty in the estimates.

## Future directions

The costs and outcomes associated with PCC revealed as part of this analysis also demonstrate the potential for PCC to further strain the Canadian healthcare system. Over the coming years, individuals with PCC will need to access the healthcare system, increasing demand for healthcare labour and other health resources (e.g. diagnostic imaging, pharmaceuticals, PCC treatments). Therefore, future research should explore where PCC is most likely to impact the healthcare system, and where future support may be needed for this population. If they do not receive appropriate care for their long-term symptoms, there could be additional quality of life and return to work impacts; and more research estimating these outcomes is needed in Canada. Moreover, as cases of PCC become increasingly identifiable in health administrative data, future analysis could also take a more direct approach to costing healthcare utilization for those with PCC, including case-control matching, propensity-score matching or micro-costing methods. Finally, the costs and outcomes associated with PCC are unlikely to be evenly felt across the Canadian population, future analysis should focus on subpopulations that may experience disparate and unequal costs and outcomes associated with PCC.

## Conclusion

This article demonstrates the large potential health and economic burden of PCC for Canadians and the Canadian healthcare system. Revealing this healthcare cost burden highlights the importance of vaccination and other adequate infection control measures to reduce the long-term healthcare costs. Moreover, the results presented here provides a timely and convenient data source for economic evaluations of COVID-19 prevention programs.

## Appendix A Search strategy

We conducted a rapid literature review on PubMed/MEDLINE. We first searched the probability of developing post COVID-19 condition (PCC) following COVID-19 infection. Then, we identified symptoms and conditions most commonly associated with PCC. Once we identified the most common symptoms of PCC, we searched the probabilities, health care costs and quality-adjusted life years (QALYs) associated with each of those symptoms. Finally, we searched the total number of PCC cases currently in Canada. Table A1 presents our search strategy. For the costs and utilities of symptoms, the table reports search terms for only diabetes. A similar strategy was followed for each of the eight symptoms we had identified.

**Table A1:** Literature search terms

Search concepts	Search terms
Probability of developing PCC following COVID-19 infection	(Probability OR Risk OR Rate OR Likelihood) AND (“Post Covid” OR “Long Covid” OR “Persistent Covid” OR “Post-acute Sequelae of SARS-CoV-2” OR “Long-haul Covid”)
Symptoms and conditions most commonly associated with PCC, and the probability they will develop	Symptom* AND (“Post Covid” OR “Long Covid” OR “Persistent Covid” OR “Post-acute Sequelae of SARS-CoV-2” OR “Long-haul Covid”)
Probability they will develop	(Prevalence OR Incidence OR Probability OR Rate OR Risk) AND Diabetes AND (“Post Covid” OR “Long Covid” OR “Persistent Covid” OR “Post-acute Sequelae of SARS-CoV-2” OR “Long-haul Covid”)
Number of PCC cases currently in Canada	(“Prevalence OR Incidence OR Rate OR “Number of” “Total Number”) AND (“Post Covid” OR “Long Covid” OR “Persistent Covid” OR “Post-acute Sequelae of SARS-CoV-2” OR “Long-haul Covid”) AND Canada
Costs associated with each symptom	Cost* AND Diabetes
QALYs associated with each symptom	(Disutility* OR “Utility Decrement” OR “QALY Decrement” OR “QALY loss”) AND Diabetes

Abbreviations: COVID-19, coronavirus disease 2019; PCC, post COVID-19 condition; QALY, quality-adjusted life year; SARS-CoV-2, severe acute respiratory syndrome coronavirus 2

## Appendix B Scenario analysis findings

**Table B1:** Overlapping and non-overlapping cost and utility decrements per COVID-19 case

Scenario	Vaccination status	Outcomes
**COVID-19 case outcomes non-overlapping scenario**		
PCC cost per COVID-19 case (CAD)	Unvaccinated	4,377
	1 dose	3,141
	2 doses	1,822
	3 doses	1,675
PCC QALY decrement per COVID-19 case	Unvaccinated	0.12
	1 dose	0.09
	2 doses	0.05
	3 doses	0.05
**COVID-19 case outcomes overlapping scenario**		
PCC cost per COVID-19 case (CAD)	Unvaccinated	7,340
	1 dose	5,268
	2 doses	3,055
	3 doses	2,809
PCC QALY decrement per COVID-19 case	Unvaccinated	0.21
	1 dose	0.15
	2 doses	0.09
	3 doses	0.08

Abbreviations: CAD, Canadian; COVID-19, coronavirus disease 2019; PCC, post COVID-19 condition; QALY, quality-adjusted life year
